# Single-baseline virtual progression modeling of Crowe type I–III developmental dysplasia of the hip: a finite element study of stress-location migration

**DOI:** 10.3389/fbioe.2026.1899897

**Published:** 2026-08-19

**Authors:** Heng Zhang, Xuanhao Zhang, Jiajun Wu, Boyu Long, Yixuan Li, Wenze Zhang, Yu Chen, Bowen Li

**Affiliations:** 1 Department of Orthopedics, The First Affiliated Hospital of Bengbu Medical University, Anhui Key Laboratory of Tissue Transplantation, Bengbu Medical University, Bengbu, Anhui, China; 2 The First School of Clinical Medicine, Bengbu Medical University, Bengbu, Anhui, China

**Keywords:** biomechanics, crowe classification, developmental dysplasia of the hip, finite element analysis, stress-location migration, virtual progression modeling

## Abstract

**Background:**

Developmental dysplasia of the hip alters acetabular morphology and load transfer, but classification-related changes in local stress distribution remain incompletely understood.

**Methods:**

A Crowe type I finite element model was directly reconstructed from one patient’s pelvic computed tomography data. Crowe type II and III models were subsequently generated from the same anatomical baseline as standardized, classification-consistent virtual morphologies rather than independent patient-specific cases. This single-baseline, internally controlled design was used to reduce inter-individual anatomical variability and evaluate morphology-related changes in stress distribution and load-transfer pathways. A simplified 600 N axial load was applied to compare peak von Mises stress and stress distribution in the acetabular-side pelvic bone, acetabular/pseudoacetabular cartilage, proximal femur, and femoral head cartilage. To assess the influence of the simplified loading assumption, an additional loading-sensitivity analysis was performed using a 1,888 N resultant force inclined medially by 16° from the global vertical direction in the frontal plane.

**Results:**

Peak stress within the acetabular-side pelvic bone region increased from Crowe type I to III (2.41, 5.79, and 6.71 MPa, respectively), whereas acetabular/pseudoacetabular cartilage stress peaked in type II (3.54 MPa). Femoral head cartilage stress increased progressively from 1.23 MPa in type I to 2.08 MPa in type II and 3.42 MPa in type III. High-stress regions migrated from the superior true acetabulum to the true acetabulum–pseudoacetabulum transition region and then to the localized pseudoacetabular load-bearing region. Under the alternative loading scenario, the principal classification-dependent stress relationships and pelvic-side stress-location migration pattern were maintained. Material-property sensitivity analyses produced peak-stress changes of no more than 2.46% and similarly preserved these patterns.

**Conclusion:**

Across the standardized Crowe type I–III virtual models, increasing classification severity was associated not only with changes in stress magnitude but also with spatial migration of the principal load-bearing regions. These findings provide an internally controlled biomechanical reference for understanding classification-related load-transfer patterns but should not be interpreted as patient-specific predictions or population-level stress thresholds. Independent patient datasets are required to validate these hypothesis-generating observations before clinical application.

## Background

Developmental dysplasia of the hip (DDH) is characterized by abnormal development of the acetabulum and proximal femur. In skeletally mature patients, DDH may manifest as acetabular shallowness, reduced femoral head coverage, subluxation, or complete dislocation, and it is an important risk factor for cartilage degeneration and hip osteoarthritis (Delgado-Arellanes et al., 2024; Riedstra et al., 2025). These morphological abnormalities can alter the mechanical environment of the hip by changing joint congruency, contact area, and load-transfer pathways. The proximal femoral load-bearing framework is organized through the primary vertical trabecular system, calcar femorale, and medial cortex, with complementary tensile support from the horizontal trabecular groups and lateral cortex (Shah et al., 2025). In DDH, altered joint contact may therefore influence stress transmission not only across the articular surfaces but also through the trabecular and cortical load-transfer pathways of the proximal femur. The Crowe classification is widely used to describe the severity of femoral head displacement and to support clinical assessment in DDH (Crowe et al., 1979). However, a displacement-based classification does not directly describe the local mechanical environment of the hip. Patients with different Crowe types may have distinct acetabular coverage, femoral head position, pseudoacetabular morphology, and tissue-specific stress concentration patterns (Martz et al., 2024). Therefore, understanding how stress distribution changes across Crowe types may provide additional biomechanical information for classification-related assessment.

Finite element analysis (FEA) has been widely used to investigate the biomechanical characteristics of normal and dysplastic hips. Previous studies have shown that acetabular undercoverage, pelvic tilt, altered acetabular geometry, and surgical correction can influence joint contact pressure and stress distribution (Incze-Bartha et al., 2024; Liu et al., 2023; Chen et al., 2022; Kitamura et al., 2021; Incze-Bartha et al., 2023; Tachibana et al., 2024; Araujo-Monsalvo et al., 2024; Aitken et al., 2024; Qu et al., 2024). However, most available studies have focused on stress magnitude, surgical reconstruction, or patient-specific correction planning. Less attention has been given to whether the anatomical location of high-stress regions changes systematically across Crowe classifications.

To our knowledge, no prior finite element study has used a single-baseline virtual progression strategy to evaluate how high-stress regions shift systematically across standardized Crowe type I–III morphologies under identical anatomical and modeling conditions. In contrast to direct comparisons among independent patient-specific models, which may be influenced by inter-individual anatomical variability, the present study used a standardized virtual progression strategy based on a single CT-derived Crowe type I model. By generating Crowe type II and III morphologies from the same anatomical baseline, this approach allowed controlled evaluation of how standardized classification-related morphological differences may alter stress distribution and load-transfer pathways. Under a standardized static baseline loading condition, we compared stress magnitude and high-stress region location in the acetabular-side pelvic bone, acetabular/pseudoacetabular cartilage, proximal femur, and femoral head cartilage. We hypothesized that increasing Crowe classification severity across the standardized virtual models would be associated with spatial migration of high-stress regions from the true acetabulum toward the pseudoacetabular load-bearing region. This modeling strategy was intended to isolate the biomechanical effects of predefined morphology changes and was not designed to reproduce the natural longitudinal progression of DDH in an individual patient.

## Methods

### Study design and ethics

This was a finite element modeling study based on pelvic computed tomography (CT) data from one patient with Crowe type I DDH. The imaging data were used to directly construct the Crowe type I hip model and served as the anatomical basis for standardized construction of Crowe type II and III models.

The patient was male, with a height of 176 cm and a body weight of 70 kg. This study was approved by the Medical Ethics Committee of Bengbu Medical University (approval number: 2025062). Written informed consent was obtained from the participant and family members.

The Crowe type I model was directly reconstructed from the CT data of one patient and therefore represented the only directly subject-specific model in the study. Crowe type II and III models were generated through standardized morphological modification of this common anatomical baseline rather than from independent patient-specific CT datasets. The resulting models should therefore be interpreted as classification-consistent virtual morphological scenarios designed for internally controlled biomechanical comparison. They were not intended to represent the full anatomical heterogeneity of clinical Crowe type II and III populations or the natural longitudinal progression of an individual patient. By maintaining a common baseline anatomy and identical material, contact, boundary, and loading settings, this strategy reduced inter-individual variability and isolated the biomechanical effects of the predefined morphological modifications.

### CT data acquisition and software

Pelvic CT plain scanning and three-dimensional reconstruction were performed using a 64-slice spiral CT scanner (Philips, Netherlands) with a slice thickness of 0.5 mm. The workstation was a Dell laptop computer equipped with an Intel Core i5-11400H processor, 24 GB memory, a 1.38 TB hard disk, and a 4 GB graphics card.

The software used in this study included Mimics 21.0 and 3-matic 13.0 (Materialise, Belgium), Geomagic Wrap 2021 (3D Systems, United States), SolidWorks 2022 (Dassault Systèmes, France), and ANSYS Workbench 2025 R2 (ANSYS, United States).

## Preprocessing of CT data

**FIGURE 1:**
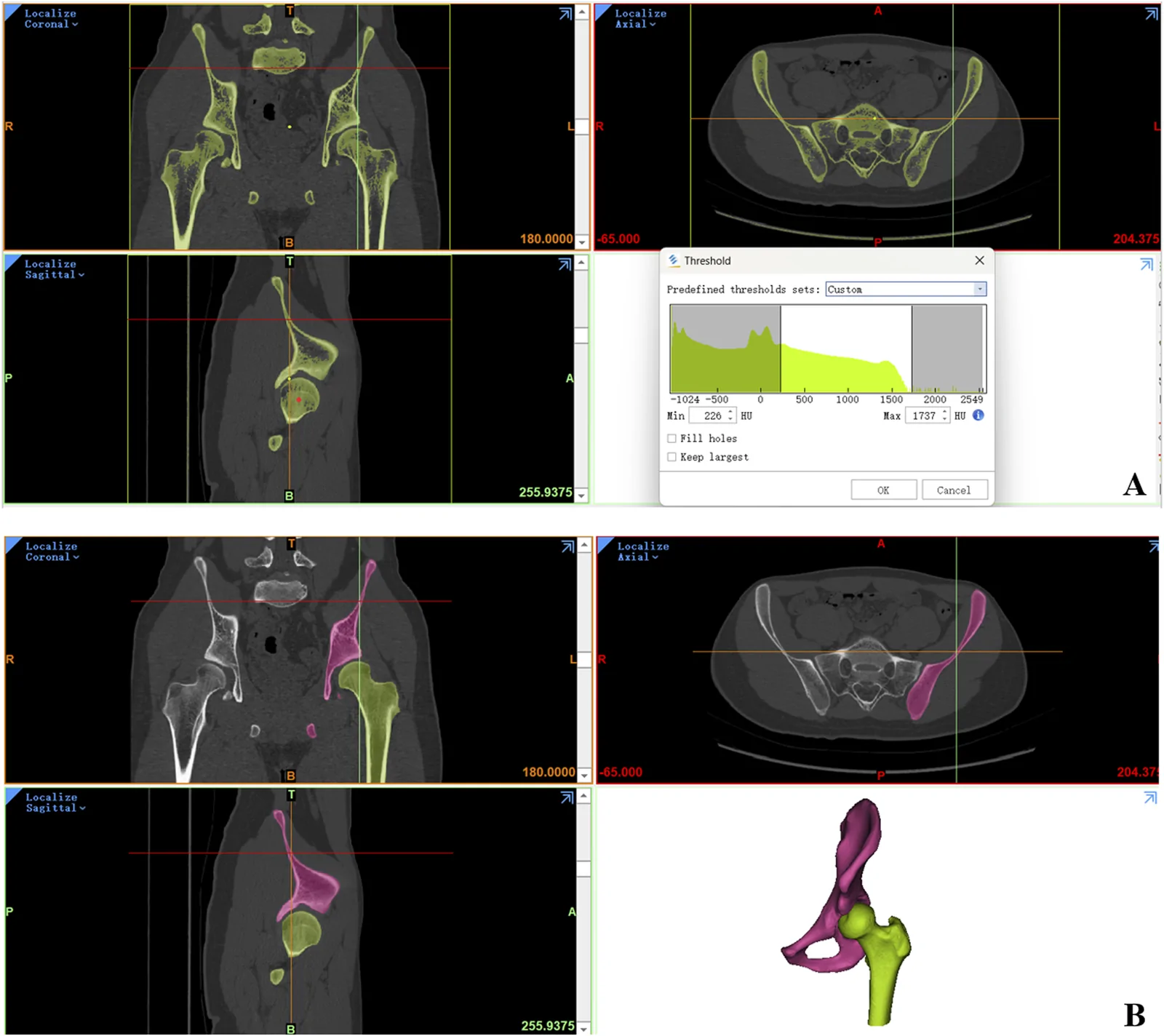
Original image segmentation and bony three-dimensional reconstruction. **(A)** Import of the original CT images. **(B)** Segmentation of the pelvis and femur with initial three-dimensional reconstruction.

Thin-slice hip CT DICOM data were imported into Mimics 21.0. The threshold range for bone tissue was set at 226–1737 HU. Region growing, mask editing, and slice-by-slice manual correction were used to segment the pelvis and femur. Irrelevant structures were removed, and the initial three-dimensional bony model was reconstructed. After smoothing, the bony model was exported in STL format for cartilage construction and finite element modeling (Figure 1).

### Construction of the Crowe type I DDH model

The preprocessed Crowe type I model from Mimics was imported into 3-matic 13.0. Cartilage boundaries were outlined on the femoral head and acetabular articular surface regions. Femoral head cartilage and acetabular cartilage were constructed separately.

Based on previous anatomical, MRI, and CT arthrography studies of hip cartilage thickness, femoral head cartilage thickness was simplified as 2.5 mm and acetabular cartilage thickness as 2.0 mm (Nakanishi et al., 2001; Wyler et al., 2007; Steppacher et al., 2021). These thickness values were applied uniformly across the corresponding articular surfaces and were maintained unchanged in all three Crowe models. This standardized cartilage representation was used to isolate the effects of the predefined osseous morphological and contact-geometry changes under otherwise identical modeling conditions. The available plain CT dataset did not provide a direct basis for reconstructing regional cartilage-thickness variation, focal cartilage defects, or degeneration-related cartilage loss. Accordingly, the cartilage models were intended for controlled biomechanical comparison rather than patient-specific reproduction of cartilage morphology or disease severity. The completed bone and cartilage models were imported into Geomagic Wrap 2021 for mesh repair, surface smoothing, hole filling, and solidification to obtain the solid Crowe type I DDH model.

### Construction of the Crowe type II DDH model

The preliminary Crowe type I model was imported into 3-matic 13.0, and femoral head height was measured as 2 cm. According to the Crowe classification, type II displacement corresponds to a superior migration distance of 50%–75% of the femoral head height (Crowe et al., 1979). Because the reconstructed femoral head height was 20 mm, a standardized 10-mm superior translation, corresponding to 50% of the femoral head height and the lower boundary of the Crowe type II displacement range, was selected to generate the Crowe type II virtual morphology. The translated femur and pelvic models were re-imported into Mimics. Boolean subtraction between the pelvic bone and the superiorly translated femur was performed to obtain the preliminary type II pelvic model.

For acetabular modification, the superior acetabular rim was manually edited slice by slice in the coronal view using Edit Masks to reduce the lateral center-edge angle to 7°. In the transverse view, the anterior and posterior acetabular walls were modified slice by slice to increase acetabular anteversion to 25°.

For femoral modification, the translated femoral model was imported into 3-matic. A cutting plane was created at the head-neck junction to separate the femoral head from the shaft. The femoral head was rotated to increase femoral anteversion to 31° and translated inferiorly to shorten the femoral neck. Boolean operations were then used to fuse the femoral head and shaft, followed by refinement and smoothing.

**FIGURE 2:**
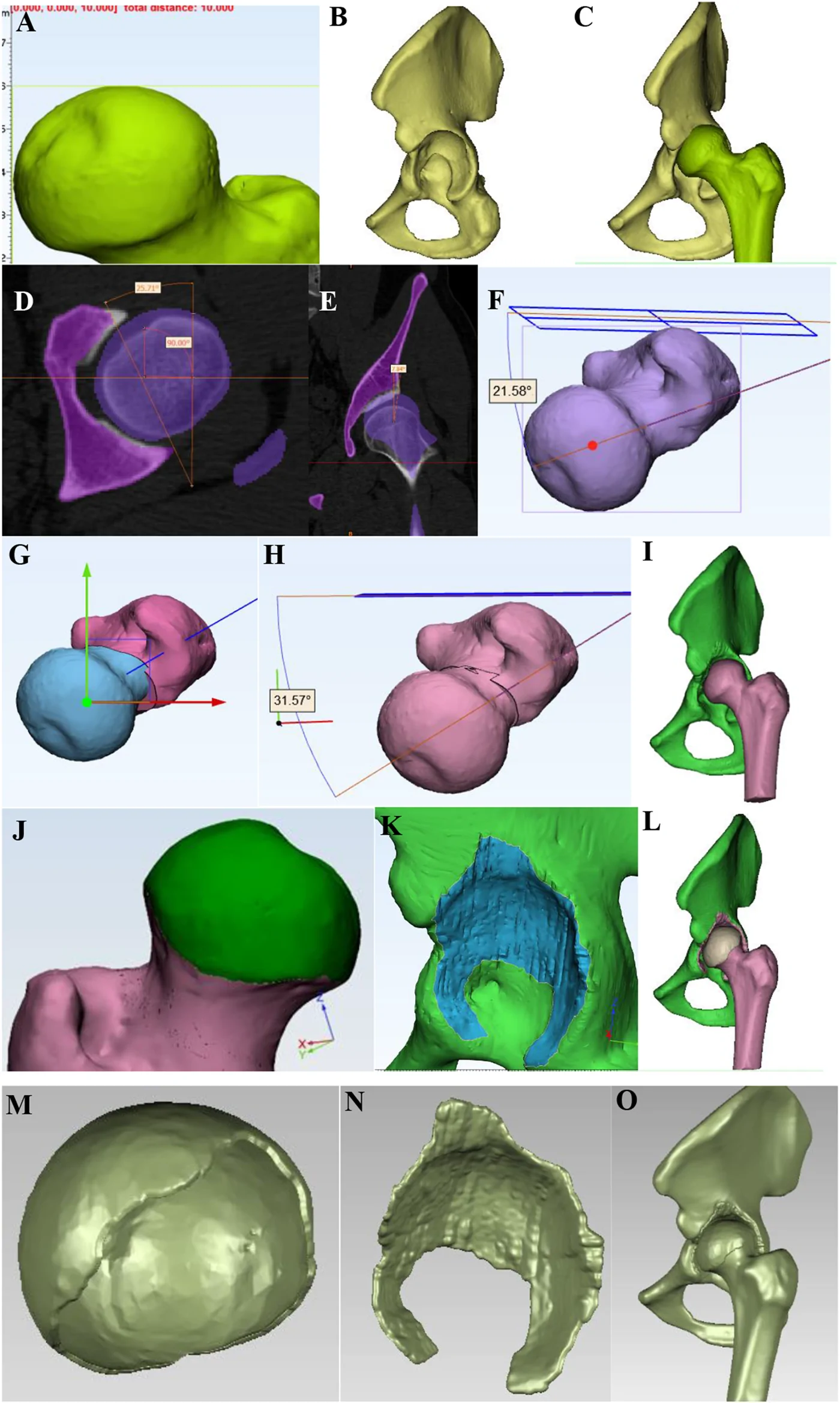
Workflow for constructing the Crowe type II DDH bone-cartilage model. **(A)** Superior translation of the femur to simulate dislocation. **(B,C)** Preliminary type II acetabular model obtained by Boolean subtraction. **(D)** Modification of acetabular anteversion. **(E)** Modification of the center-edge angle. **(F)** Measurement of original femoral anteversion. **(G)** Rotation of the femoral head to increase femoral anteversion. **(H)** Inferior repositioning of the femoral head and reconstruction with the femoral shaft. **(I)** Final bony model after refinement and smoothing. **(J,K)** Femoral head and acetabular cartilage construction. **(L)** Type II model after cartilage construction. **(M–O)** Final solidified bone-cartilage model.

Cartilage was constructed by outlining the femoral cartilage coverage boundary on the femoral head surface using the Create Curve function. Cartilage and bone surfaces were separated using Split Surfaces by Curves, and a femoral cartilage model with a thickness of 2.5 mm was created using Create Base. The same approach was applied to the true acetabulum and pseudoacetabular regions to construct acetabular/pseudoacetabular cartilage with a thickness of 2.0 mm. The completed model was imported into Geomagic Wrap 2021 for preprocessing, noise reduction, smoothing, automatic surfacing, patch repair, grid repair, surface fitting, and solidification (Figure 2).

### Construction of the Crowe type III DDH model

**FIGURE 3:**
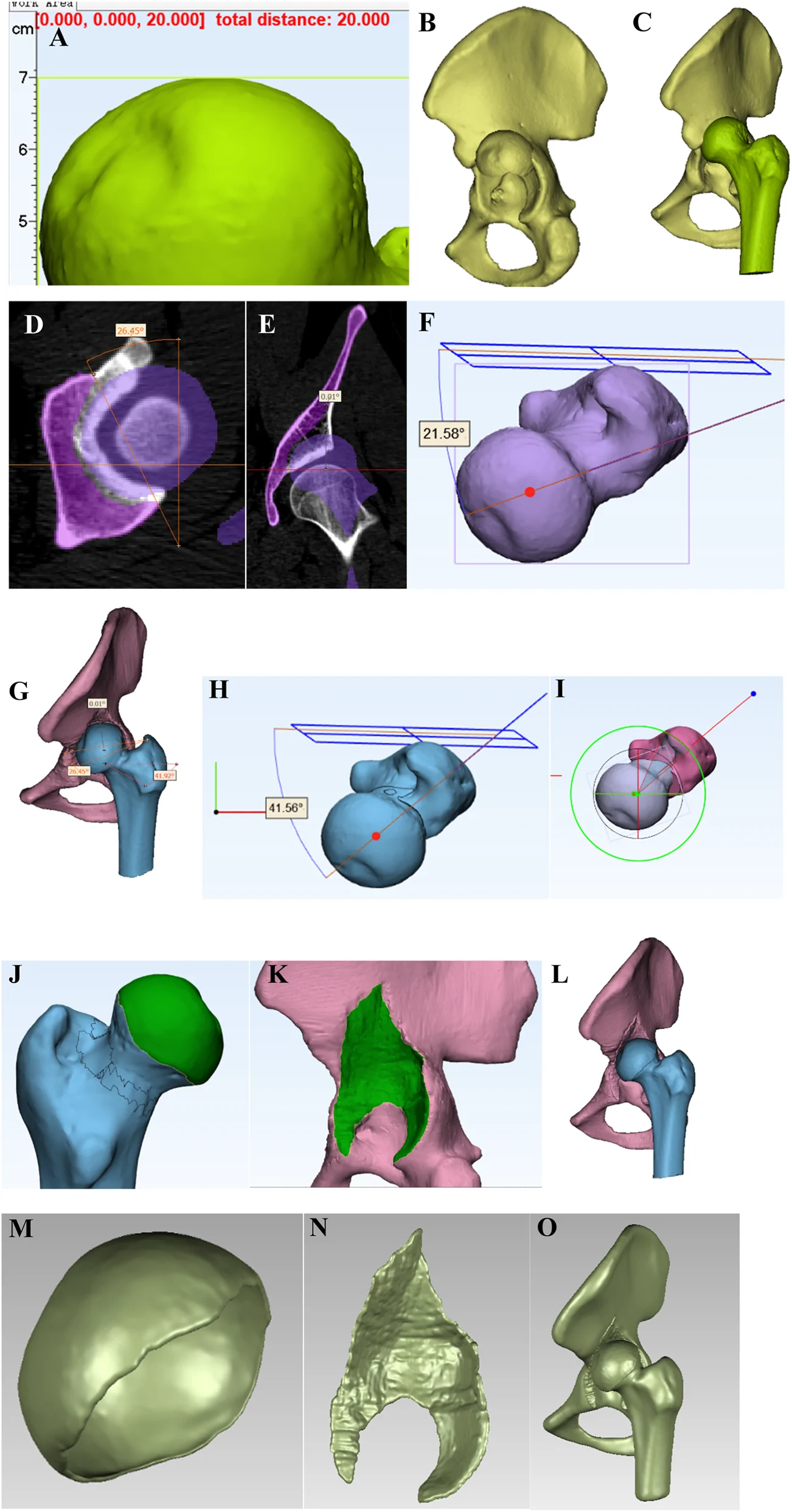
Workflow for constructing the Crowe type III DDH bone-cartilage model. **(A)** Superior translation of the femur to simulate dislocation. **(B,C)** Preliminary type III acetabular model obtained by Boolean subtraction. **(D)** Modification of acetabular anteversion. **(E)** Modification of the center-edge angle. **(F)** Measurement of original femoral anteversion. **(G)** Rotation of the femoral head to increase femoral anteversion. **(H)** Inferior repositioning of the femoral head and reconstruction with the femoral shaft. **(I)** Final bony model after refinement and smoothing. **(J,K)** Femoral head and acetabular cartilage construction. **(L)** Type III model after cartilage construction. **(M–O)** Final solidified bone-cartilage model.

The Crowe type III model was generated using the same standardized workflow used for the type II model, including bony morphological modification, cartilage construction, and solidification. For the Crowe type III morphology, the femur was translated superiorly by 20 mm, corresponding to 100% of the reconstructed femoral head height and the upper boundary of the Crowe type III displacement range (Crowe et al., 1979). The 10-mm incremental translation from type II to type III was selected to provide a reproducible stepwise virtual displacement series. The acetabular lateral center-edge angle was reduced to 0°, acetabular anteversion was increased to 26°, and femoral anteversion was increased to 41°. After the same cartilage construction and solidification procedures, the solid Crowe type III DDH model was obtained (Figure 3).

### Rationale for morphological parameter selection

The morphological parameters were selected to generate standardized, classification-consistent virtual scenarios rather than to reproduce Crowe-specific population means. Superior femoral translations of 10 and 20 mm were determined from the reconstructed femoral head height of 20 mm and the Crowe displacement criteria (Crowe et al., 1979). Lateral center-edge angle targets of 7° and 0° were selected to represent marked and near-complete lateral acetabular undercoverage, respectively, within the range reported for severe hip dysplasia (Kitamura et al., 2021; Fujii et al., 2016). Femoral anteversion was increased to 31° and 41° to reflect the documented tendency toward increased and highly variable femoral anteversion with increasing DDH severity (Hu et al., 2022; Chen et al., 2024; Boughton et al., 2019). Published CT studies have reported mean femoral anteversion values of approximately 28.6°–33.3° in Crowe type II hips and 34.9°–35.5° in Crowe type III hips (Hu et al., 2022; Chen et al., 2024). Sex-stratified CT data have also reported femoral anteversion values of approximately 44° in female Crowe type III hips, illustrating the substantial clinical variability of this parameter (Boughton et al., 2019). Acetabular anteversion targets of 25° and 26° were selected as upper-range dysplastic scenarios within the broad variability reported in CT-based morphological studies, rather than as Crowe type-specific mean values (Chen et al., 2024; Gao et al., 2025). Accordingly, these parameters should be interpreted as predefined modeling targets used for controlled biomechanical comparison and not as diagnostic thresholds or population averages. The main modeling parameters are summarized in Table 1.

**TABLE 1:** Morphological parameters used for standardized model construction.

Crowe classification	Superior translation of femur	Lateral center-edge angle	Acetabular anteversion	Femoral anteversion
Type I	Direct CT-based model	Original CT-based morphology	Original CT-based morphology	Original CT-based morphology
Type II	1 cm	7°	25°	31°
Type III	2 cm	0°	26°	41°

The Crowe type II and III models were generated through standardized morphological modification of the same Crowe type I anatomical baseline. Superior translation distances were determined according to the reconstructed femoral head height and the Crowe displacement criteria (Crowe et al., 1979). Center-edge angle and anteversion values were predefined modeling targets informed by published radiographic and CT-based morphological ranges (Kitamura et al., 2021; Fujii et al., 2016; Hu et al., 2022; Chen et al., 2024; Boughton et al., 2019; Gao et al., 2025). They were not direct measurements from separate patients, Crowe-specific population means, or diagnostic thresholds.

### Material properties

The solidified models were imported into SolidWorks 2022 to verify assembly relationships and then imported into ANSYS Workbench 2025 R2. Based on material parameters used in previous orthopedic biomechanical studies, all modeled tissues were assigned isotropic, linear elastic properties (Table 2).

**TABLE 2:** Material properties assigned to the finite element models.

Tissue/component	Young’s modulus (MPa)	Poisson’s ratio	Material assumption
Pelvic and femoral cortical bone	17,000	0.30	Isotropic, linear elastic
Femoral head and acetabular cartilage	5	0.46	Isotropic, linear elastic

### Contact relationships

Contact relationships were defined consistently across all Crowe type I–III models. The interfaces between bone and cartilage were defined as bonded contact to maintain attachment between the cartilage layer and the underlying bony surface. The contact between opposing cartilage surfaces was defined using a no-separation contact setting to allow load transfer while preventing artificial separation under the simplified static loading condition. The same contact definitions were used in all models to ensure comparability across Crowe classifications.

### Boundary conditions and baseline loading settings

**FIGURE 4:**
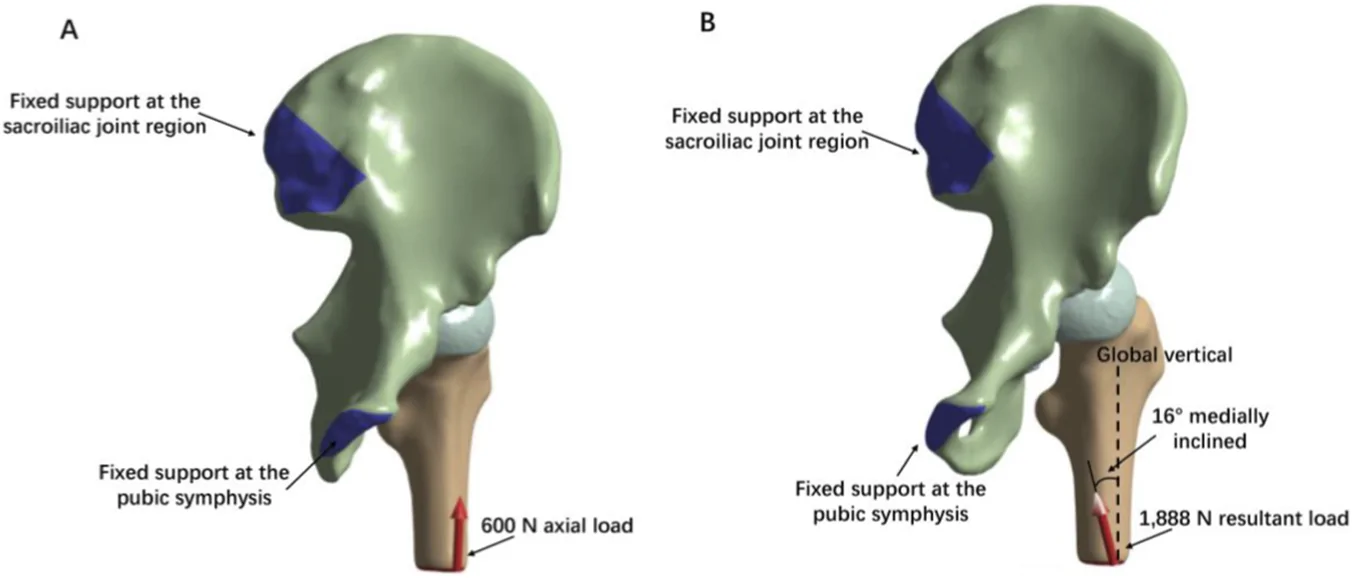
Boundary constraints and loading conditions applied to the finite element models. **(A)** Baseline loading condition with a 600 N axial load applied to the distal femoral section. **(B)** Alternative loading condition with a 1,888 N resultant force inclined medially by 16° from the global vertical direction in the frontal plane. Fixed constraints were applied to the sacroiliac joint region and pubic symphysis under both loading conditions. Each loading condition was applied consistently across the Crowe type I–III models.

A simplified static loading condition was used as the baseline scenario. Identical boundary and loading settings were applied to all Crowe type I–III models to facilitate internally controlled comparison. Fixed constraints were applied to the sacroiliac joint region and pubic symphysis to represent pelvic stabilization. A 600 N axial compressive load was applied to the distal femoral section (Figure 4).

The selected magnitude represented a body-weight-level external load and was informed by previous simplified hip finite element studies. Zhang et al. applied a 567 N load, corresponding to approximately 81% of body weight in a 70-kg subject, to simulate static single-leg weight bearing, whereas Rudman et al. applied a 600 N body-weight load through the base of the femoral shaft in a simplified one-legged stance model (Zhang et al., 2020; Rudman et al., 2006). However, the loading locations, constraints, and inclusion of muscle and ligament forces differed among these models.

Therefore, the 600 N condition in the present study was interpreted as a standardized body-weight-scale baseline load for internally controlled comparison rather than as the complete physiological hip joint reaction force during single-leg stance or gait. The baseline analysis consequently focused on relative differences in stress distribution among the standardized Crowe models rather than on absolute physiological stress values.

### Alternative loading-scenario sensitivity analysis

Because a body-weight-level external load does not account for the amplification of hip joint force produced by muscle recruitment and moment equilibrium, an alternative higher-magnitude oblique loading scenario was applied to all three Crowe models to assess the sensitivity of the principal stress-distribution patterns to the baseline loading assumption. For the 70-kg subject, a resultant force of 1,888 N, corresponding to approximately 2.75 times body weight, was applied to the distal femoral section. The force was inclined medially by 16° from the global vertical direction in the frontal plane, with global force components of −521 N, 0 N, and 1,815 N along the X, Y, and Z-axes, respectively. The selected magnitude was used as a higher-load sensitivity condition informed by the physiological order of magnitude reported in *in vivo* hip-contact-force studies, whereas the loading direction was informed by published observations indicating that the frontal-plane hip joint resultant is oriented approximately 16° from the vertical (Kotzar et al., 1991; Bergmann et al., 2001; Lee et al., 2007). Geometry, mesh, material properties, cartilage thickness, contact definitions, pelvic constraints, and solver settings were maintained unchanged. This alternative scenario was used to test the sensitivity of the principal findings to load magnitude and direction. It was not intended to represent a complete muscle-driven or dynamic simulation of single-leg stance or gait.

### Material-property sensitivity analysis

To quantify uncertainty associated with the simplified homogeneous material assignments, separate one-factor-at-a-time material-property sensitivity analyses were performed under the baseline 600 N loading condition. The Young’s modulus of bone was varied by ±20% from the baseline value of 17,000 MPa, resulting in values of 13,600 and 20,400 MPa, while the cartilage modulus was maintained at 5 MPa. The Young’s modulus of cartilage was independently varied by ±20% from the baseline value of 5 MPa, resulting in values of 4 and 6 MPa, while the bone modulus was maintained at 17,000 MPa.

Poisson’s ratios were maintained at 0.30 for bone and 0.46 for cartilage. Geometry, mesh, cartilage thickness, contact definitions, boundary constraints, loading conditions, and solver settings were maintained unchanged. The analyses were performed for all Crowe type I–III models. Peak von Mises stresses and the anatomical locations of the principal high-stress regions were compared with those obtained using the corresponding baseline material properties.

### Numerical verification and modeling consistency

Numerical verification was assessed through mesh-independence analysis, while modeling consistency was ensured by maintaining identical material properties, contact definitions, boundary conditions, and solver settings across the three Crowe models. These procedures were used to evaluate numerical stability and internal comparability but were not considered substitutes for experimental model validation. All models were meshed using tetrahedral solid elements. Mesh independence was evaluated using the Crowe type III model, which had the most complex geometry and contact relationships among the three models. Coarse, medium, and fine meshes were generated by varying the global mesh density while keeping material properties, contact relationships, boundary conditions, and loading conditions unchanged. Peak von Mises stresses of the pelvic bone, acetabular/pseudoacetabular cartilage, proximal femur, and femoral head cartilage were compared across mesh levels. Mesh convergence was considered acceptable when the relative change in peak von Mises stress from the medium to fine mesh was less than 5%. The element and node numbers, together with peak stress values for each mesh level, are provided in Supplementary Table S1.

### External benchmarking and scope of validation

No patient-matched experimental measurements were available for the source subject; therefore, direct experimental validation of the present finite element model was not possible. Instead, the CT-derived Crowe type I baseline model was externally benchmarked against published experimental observations and previously validated hip finite element studies. The anatomical location of acetabular-side stress concentration was compared qualitatively with experimental pressure-mapping findings obtained under reduced acetabular coverage, while the proximal femoral load-transfer pattern was compared with published anatomical and finite element evidence regarding load transmission through the femoral neck, calcar femorale, and medial cortex (Shah et al., 2025; Tachibana et al., 2024; Anderson et al., 2008; Henak et al., 2014).

Published cadaveric validation studies have demonstrated that specimen-specific hip finite element models can reproduce experimentally measured cartilage contact-stress patterns when specimen geometry, loading conditions, and experimental boundary conditions are closely matched (Anderson et al., 2008; Henak et al., 2014). However, because the published studies differed from the present study in specimen anatomy, loading conditions, material formulations, contact definitions, and outcome variables, the comparison was used only to assess anatomical and biomechanical plausibility. No numerical validation error or claim of direct experimental validation was made.

The Crowe type II and III models were standardized virtual morphologies generated from the same Crowe type I anatomical baseline and were not validated against independent patient-specific CT datasets. They were therefore interpreted as controlled classification-consistent modeling scenarios rather than validated representations of clinical Crowe type II and III populations.

### Anatomical definition of the true acetabulum–pseudoacetabulum transition region

Following the anatomical relationship described in the Hartofilakidis classification, the false or pseudoacetabulum was considered to overlap the true acetabulum when the inferior margin of the pseudoacetabular support region extended to or below the superior margin of the native true acetabulum (Hartofilakidis et al., 1996; Hartofilakidis et al., 2008). Accordingly, the true acetabulum–pseudoacetabulum transition region was defined as the contact or overlap region between the superior margin of the native true acetabulum and the inferior margin of the modeled pseudoacetabulum.

In the standardized coronal view, the superior limit of the true acetabulum and the most inferior point of the pseudoacetabulum were used as the principal anatomical landmarks for localizing this region, consistent with the radiographic landmarks described by Hartofilakidis et al. (Hartofilakidis et al., 2008). The corresponding region was then identified on the contiguous three-dimensional bony and cartilage surfaces for interpretation of the stress contour maps. In this manuscript, the term “transition region” is used as a model-specific descriptive label for this established true–false acetabular contact or overlap relationship, rather than as a separate universally standardized anatomical structure.

### Outcome measures and statistical analysis

The primary outcome measures were peak von Mises stress and stress distribution characteristics in the acetabular-side pelvic bone, acetabular/pseudoacetabular cartilage, proximal femur, and femoral head cartilage. Von Mises stress was selected as a common scalar measure to enable consistent comparison of the magnitude and spatial location of multiaxial stress states across the four modeled structures under identical modeling conditions. For cartilage, it was used as a comparative descriptor of stress concentration rather than as a direct measure of contact pressure, tissue damage, degeneration, or failure. Stress contour plots were exported for comparative analysis. For visualization, identical contour limits, color scales, viewing orientations, and display settings were applied across the Crowe type I–III models for each tissue under the same loading condition. Because stress magnitudes differed substantially among tissue types, tissue-specific common scales were used rather than a single scale across all structures.

No inferential statistical testing or confidence-interval estimation was performed because the study comprised a deterministic series of standardized models rather than a sample of independent subjects. Model uncertainty was instead assessed using loading-scenario and material-property sensitivity analyses. Peak von Mises stress values were extracted from ANSYS Workbench 2025 R2 and compared descriptively across Crowe type I–III models. Stress distribution was assessed qualitatively using stress contour plots, with emphasis on anatomical location and spatial migration of high-stress regions.

For the loading-sensitivity analysis, peak von Mises stress, relative changes in peak stress, and the anatomical locations of the principal high-stress regions were compared between the baseline 600 N axial condition and the alternative 1,888 N oblique condition. Particular attention was given to whether the classification-related stress-location migration pattern remained present under the alternative loading scenario.

For the material-property sensitivity analyses, peak stress values, relative changes from the corresponding baseline material condition, classification-dependent stress ordering, and the anatomical locations of the principal high-stress regions were compared across the ±20% bone- and cartilage-modulus scenarios.

## Results

### Model construction and mesh independence

Three-dimensional finite element models of Crowe type I–III DDH were successfully constructed. The Crowe type I model was directly generated from CT-based reconstruction, while the type II and III models were generated through standardized morphological modification of the type I model. The same material properties, contact relationships, boundary conditions, and loading settings were applied to all three models.

Mesh independence analysis showed that peak von Mises stresses of the main structures stabilized with progressive mesh refinement. When the mesh was refined from the medium to the fine level, the relative changes in peak stress were 1.51% for pelvic bone, 1.73% for acetabular/pseudoacetabular cartilage, 1.74% for the proximal femur, and 2.05% for femoral head cartilage, all of which were below the predefined 5% threshold (Supplementary Table S1). Therefore, the medium mesh was selected for subsequent comparative analysis. Because model settings were kept consistent across Crowe classifications, differences in stress distribution were interpreted as classification-related biomechanical patterns rather than as consequences of inconsistent modeling conditions.

### External benchmarking of the Crowe type I baseline model

In the Crowe type I baseline model, the principal acetabular-side high-stress region was located near the superior acetabular rim. This distribution was qualitatively consistent with experimental pressure-mapping findings showing that reduced acetabular coverage concentrates load toward the acetabular rim (Tachibana et al., 2024). The proximal femoral high-stress region was primarily located in the femoral neck and medial load-transfer region. This pattern was also qualitatively consistent with anatomical and finite element evidence identifying the calcar femorale, primary vertical trabecular system, and medial cortex as major compressive load-transfer pathways in the proximal femur (Shah et al., 2025).

These comparisons supported the anatomical and biomechanical plausibility of the Crowe type I stress-distribution pattern. However, because the referenced studies used different species or specimens, loading and boundary conditions, material representations, and mechanical outcome measures, the observed agreement was qualitative and did not constitute direct experimental validation of the present model.

### Peak von Mises stress across Crowe type I–III models

The peak von Mises stresses of the acetabular-side pelvic bone, acetabular/pseudoacetabular cartilage, proximal femur, and femoral head cartilage in Crowe type I–III DDH models are shown in Table 3.

**TABLE 3:** Peak von Mises stress of hip structures in Crowe type I–III DDH models.

Crowe classification	Acetabular-side pelvic bone	Acetabular/pseudoacetabular cartilage	Proximal femur	Femoral head cartilage
Type I	2.41	2.08	4.48	1.23
Type II	5.79	3.54	4.37	2.08
Type III	6.71	2.31	4.61	3.42

Unit: MPa.

Peak stress within the acetabular-side pelvic bone region increased progressively from 2.41 MPa in Crowe type I to 5.79 MPa in type II and 6.71 MPa in type III. Proximal femoral peak stress was 4.48 MPa in Crowe type I, 4.37 MPa in type II, and 4.61 MPa in type III. The type I and II values were comparable, with the type I value slightly higher, whereas the highest value occurred in type III. Femoral head cartilage stress increased from 1.23 MPa in type I to 2.08 MPa in type II and 3.42 MPa in type III.

Acetabular/pseudoacetabular cartilage stress showed a different pattern. The peak stress was 2.08 MPa in type I, increased to 3.54 MPa in type II, and decreased to 2.31 MPa in type III.

### Acetabular-side pelvic bone high-stress regions migrated across the standardized Crowe type I–III models

**FIGURE 5:**
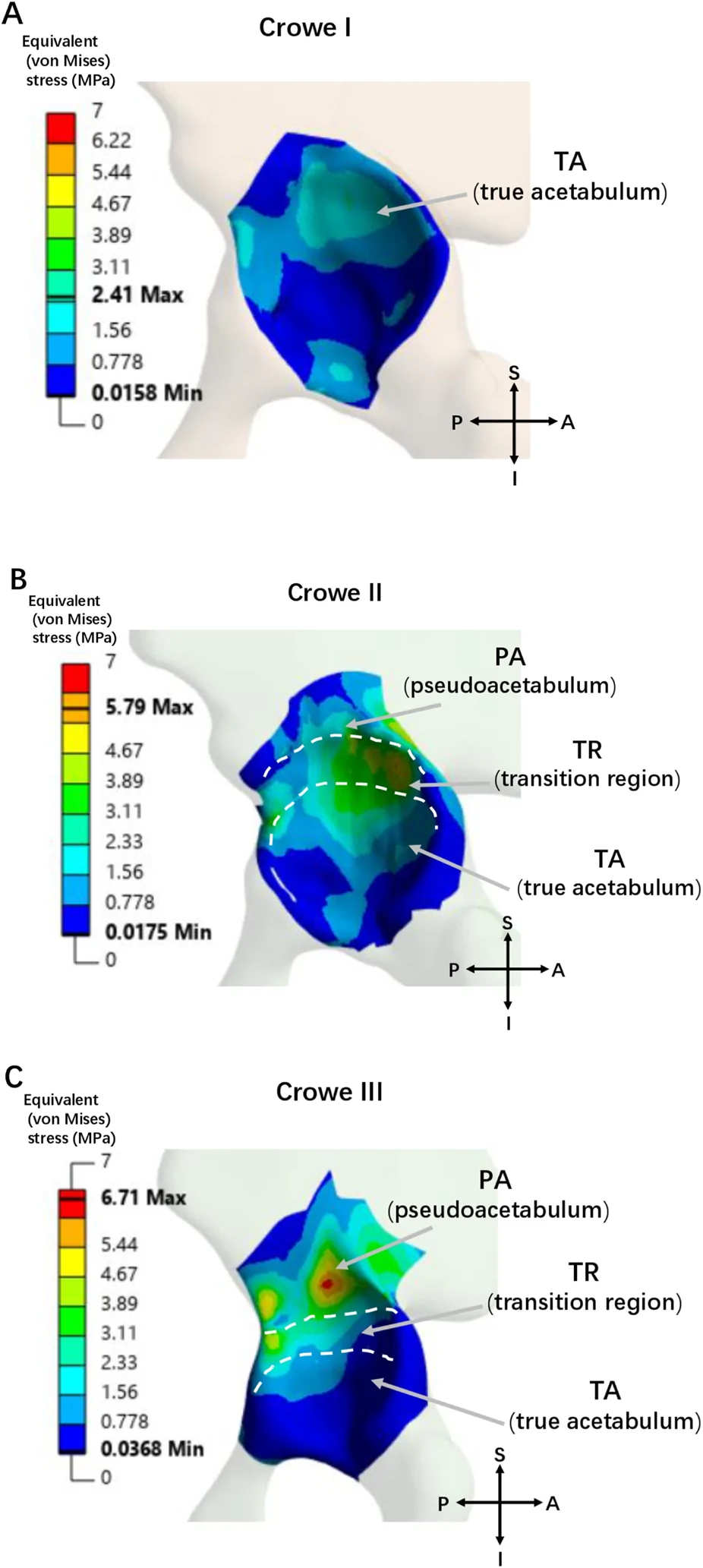
Acetabular-side pelvic bone stress distribution in Crowe type I–III DDH. **(A)** Crowe type I. **(B)** Crowe type II. **(C)** Crowe type III. The panels display the predefined acetabular-side pelvic bone region of interest. Color maps indicate von Mises stress in MPa. An identical color scale of 0–7 MPa and a standardized viewing orientation were applied to all three panels to facilitate direct comparison. Peak values refer to the predefined acetabular-side pelvic bone region rather than the whole-pelvis global maximum. Exact peak stress values are reported in Table 3. TA, true acetabulum; TR, true acetabulum–pseudoacetabulum transition region; PA, pseudoacetabulum; S, superior; I, inferior; A, anterior; P, posterior.

Within the acetabular-side pelvic bone region, the high-stress region shifted progressively with increasing Crowe classification severity. In Crowe type I, the high-stress region was mainly located in the superior load-bearing region of the true acetabulum. In type II, the high-stress region shifted to the true acetabulum–pseudoacetabulum transition region, defined as the contact or overlap region between the superior margin of the native true acetabulum and the inferior margin of the modeled pseudoacetabulum (Figure 5). In type III, the high-stress region further migrated to the localized bony support area corresponding to the pseudoacetabulum (Figure 5).

### Acetabular/pseudoacetabular cartilage stress peaked in Crowe type II

**FIGURE 6:**
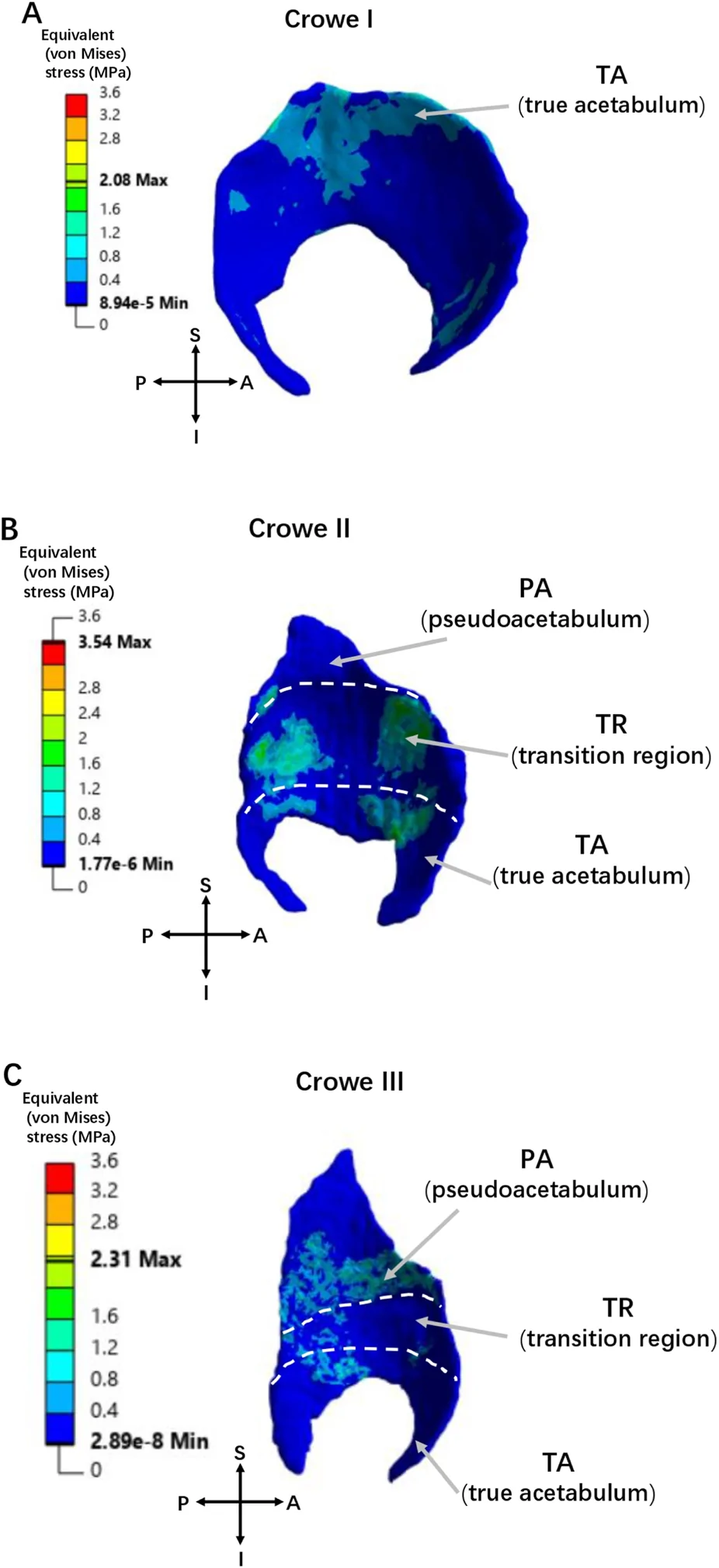
Acetabular/pseudoacetabular cartilage stress distribution in Crowe type I–III DDH. **(A)** Crowe type I. **(B)** Crowe type II. **(C)** Crowe type III. Color maps indicate von Mises stress distribution in MPa. An identical color scale of 0–3.6 MPa and a standardized viewing orientation were applied to all three panels to facilitate direct comparison. Exact peak stress values are reported in Table 3. TA, true acetabulum; TR, true acetabulum–pseudoacetabulum transition region; PA, pseudoacetabulum; S, superior; I, inferior; A, anterior; P, posterior.

Acetabular/pseudoacetabular cartilage peak stress was highest in Crowe type II. Stress contour plots showed relatively low overall cartilage stress in type I. In type II, stress was concentrated in the true acetabulum–pseudoacetabulum transition region and adjacent local contact region. In type III, stress distribution was more dispersed within the modeled pseudoacetabular cartilage region (Figure 6).

### Proximal femoral stress was highest in Crowe type III

**FIGURE 7:**
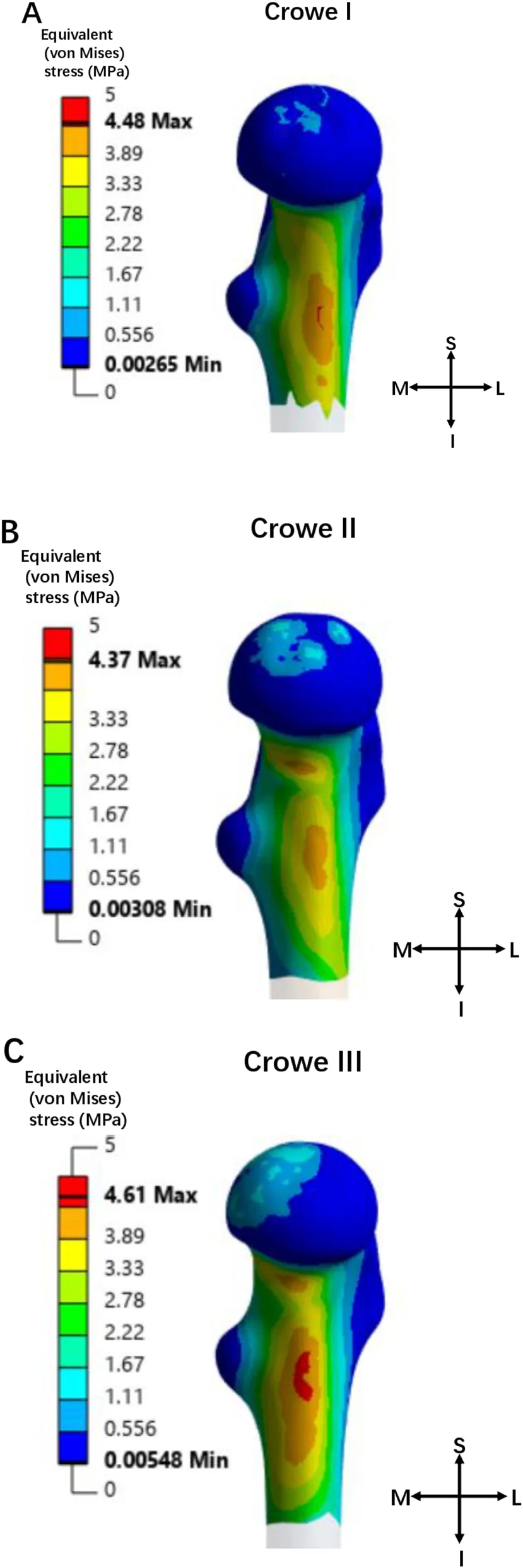
Proximal femoral stress distribution in Crowe type I–III DDH. **(A)** Crowe type I. **(B)** Crowe type II. **(C)** Crowe type III. Color maps indicate von Mises stress distribution in MPa. An identical color scale of 0–5 MPa and a standardized viewing orientation were applied to all three panels to facilitate direct comparison. Exact peak stress values are reported in Table 3. S, superior; I, inferior; M, medial; L, lateral.

Proximal femoral peak stress was highest in Crowe type III, while the type I value was slightly higher than the type II value. In type I, stress was concentrated mainly at the femoral neck base and medial proximal femur. In type II, the high-stress distribution became broader. In type III, stress was concentrated in the femoral neck and medial load-transfer region of the proximal femur (Figure 7).

### Femoral head cartilage stress concentrated at the displaced contact region

**FIGURE 8:**
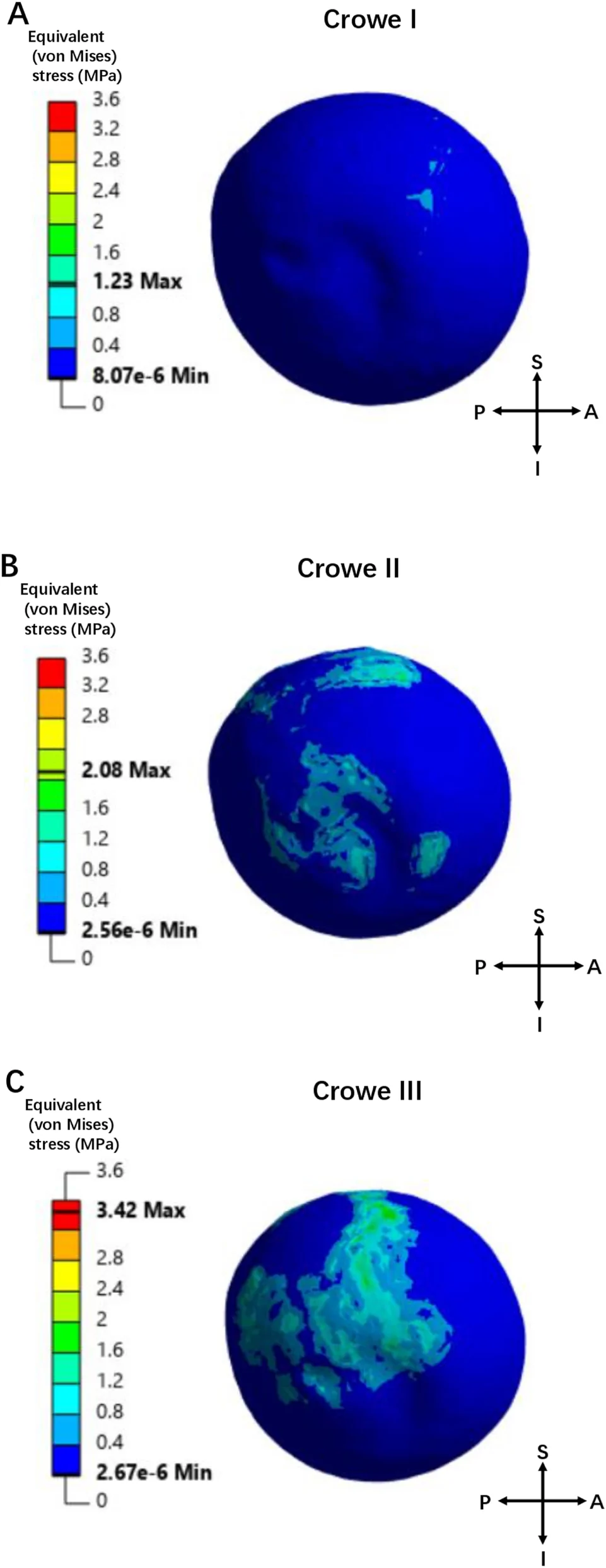
Femoral head cartilage stress distribution in Crowe type I–III DDH. **(A)** Crowe type I. **(B)** Crowe type II. **(C)** Crowe type III. Color maps indicate von Mises stress distribution in MPa. An identical color scale of 0–3.6 MPa and a standardized articular-surface viewing orientation were applied to all three panels to facilitate direct comparison. Exact peak stress values are reported in Table 3. S, superior; I, inferior; A, anterior; P, posterior.

Femoral head cartilage peak stress increased progressively from type I to type III. The high-stress region was limited to a local contact area in type I, shifted to the superolateral load-bearing region of the femoral head in type II, and concentrated at the load-bearing apex contacting the pseudoacetabulum in type III (Figure 8).

### Stress distribution maps showed classification-dependent spatial migration

Across the modeled structures, the principal high-stress regions shifted systematically with increasing Crowe classification severity in the standardized virtual models (Figures 5–8). In type I, high-stress regions were concentrated in the superior true acetabular load-bearing area and the local femoral head contact surface. In type II, high stress was concentrated in the true acetabulum–pseudoacetabulum transition region and the superolateral femoral head. In type III, high stress was located mainly in the localized pseudoacetabular bony support region and the corresponding femoral head contact apex.

These findings indicate that the main biomechanical pattern associated with increasing Crowe classification severity across the standardized models was not limited to changes in peak stress magnitude. A spatial shift of the load-bearing center was also observed.

### Alternative loading-scenario sensitivity analysis

Under the alternative 1,888 N oblique loading condition, peak stresses within the acetabular-side pelvic bone region were 7.29, 17.51, and 20.30 MPa in the Crowe type I, II, and III models, respectively. The corresponding acetabular/pseudoacetabular cartilage stresses were 6.29, 10.72, and 6.97 MPa; proximal femoral stresses were 13.55, 13.22, and 13.96 MPa; and femoral head cartilage stresses were 3.71, 6.30, and 10.34 MPa, respectively (Table 4).

**TABLE 4:** Peak von Mises stresses under the alternative 1,888 N oblique loading condition and their relative changes from the baseline condition.

Crowe classification	Acetabular-side pelvic bone	Acetabular/pseudoacetabular cartilage	Proximal femur	Femoral head cartilage
Type I	7.29 (+202.3%)	6.29 (+202.4%)	13.55 (+202.5%)	3.71 (+201.3%)
Type II	17.51 (+202.5%)	10.72 (+202.7%)	13.22 (+202.5%)	6.30 (+203.0%)
Type III	20.30 (+202.4%)	6.97 (+201.9%)	13.96 (+202.8%)	10.34 (+202.2%)

Unit: MPa. Values are peak von Mises stresses in MPa. Percentage changes relative to the corresponding baseline 600 N condition are shown in parentheses. Percentage changes were calculated using the unrounded baseline values.

Compared with the baseline 600 N condition, peak stresses increased by 201.3%–203.0% across all 12 tissue–model comparisons. The classification-dependent stress relationships were maintained under the alternative loading condition. Acetabular-side pelvic bone stress remained ordered as Crowe type I < type II < type III; acetabular/pseudoacetabular cartilage stress remained highest in type II; proximal femoral stress remained ordered as type II < type I < type III; and femoral head cartilage stress retained its progressive type I–III increase.

Stress contour comparison showed that the principal pelvic-side high-stress regions remained located at the superior true-acetabular load-bearing region in type I, the true acetabulum–pseudoacetabulum transition region in type II, and the localized pseudoacetabular support region in type III (Supplementary Figure S1). Thus, the principal pelvic-side stress-location migration pattern remained present under the alternative loading scenario.

### Material-property sensitivity analysis

Variation of the cartilage Young’s modulus by ±20% produced relative peak-stress changes ranging from −2.46% to +1.64% across the assessed tissues and Crowe models. Variation of the bone Young’s modulus by ±20% resulted in corresponding changes ranging from −2.06% to +1.99%. Across all 48 non-baseline tissue–model comparisons, the absolute relative change did not exceed 2.46% (Table 5).

**TABLE 5:** Material-property sensitivity analysis under the baseline 600 N loading condition.

Crowe type	Cartilage modulus (MPa)	Acetabular-side pelvic bone	Acetabular/pseudoacetabular cartilage	Proximal femur	Femoral head cartilage
Panel A. Cartilage Young’s modulus sensitivity					
I	4	2.4081 (−0.14%)	2.0291 (−2.45%)	4.4956 (+0.40%)	1.1997 (−2.46%)
I	5, baseline	2.4114	2.0800	4.4777	1.2300
I	6	2.4132 (+0.07%)	2.1142 (+1.64%)	4.4656 (−0.27%)	1.2426 (+1.02%)
II	4	5.7443 (−0.78%)	3.4767 (−1.79%)	4.3608 (−0.21%)	2.0640 (−0.77%)
II	5, baseline	5.7893	3.5400	4.3700	2.0800
II	6	5.8192 (+0.52%)	3.5877 (+1.35%)	4.3750 (+0.11%)	2.0976 (+0.85%)
III	4	6.7299 (+0.27%)	2.2890 (−0.91%)	4.6083 (−0.04%)	3.3953 (−0.72%)
III	5, baseline	6.7119	2.3100	4.6100	3.4200
III	6	6.6954 (−0.25%)	2.3226 (+0.55%)	4.6192 (+0.20%)	3.4358 (+0.46%)

Crowe type	Bone modulus (MPa)	Acetabular-side pelvic bone	Acetabular/pseudoacetabular cartilage	Proximal femur	Femoral head cartilage
Panel B. Bone Young’s modulus sensitivity					
I	13,600	2.4135 (+0.09%)	2.1214 (+1.99%)	4.4631 (−0.33%)	1.2461 (+1.31%)
I	17,000, baseline	2.4114	2.0800	4.4777	1.2300
I	20,400	2.4088 (−0.11%)	2.0389 (−1.98%)	4.4920 (+0.32%)	1.2047 (−2.06%)
II	13,600	5.8251 (+0.62%)	3.5968 (+1.60%)	4.3759 (+0.14%)	2.1005 (+0.99%)
II	17,000, baseline	5.7893	3.5400	4.3700	2.0800
II	20,400	5.7532 (−0.62%)	3.4896 (−1.42%)	4.3626 (−0.17%)	2.0678 (−0.59%)
III	13,600	6.6914 (−0.31%)	2.3269 (+0.73%)	4.6209 (+0.24%)	3.4402 (+0.59%)
III	17,000, baseline	6.7119	2.3100	4.6100	3.4200
III	20,400	6.7268 (+0.22%)	2.2917 (−0.79%)	4.6096 (−0.01%)	3.3991 (−0.61%)

Values are peak von Mises stresses in MPa. Percentage changes relative to the corresponding baseline material condition are shown in parentheses. Bone and cartilage Young’s moduli were varied separately, while all other modeling conditions were maintained unchanged.

The classification-dependent stress relationships were maintained under all tested material conditions. Acetabular-side pelvic bone peak stress remained ordered as Crowe type I < type II < type III. Acetabular/pseudoacetabular cartilage stress remained highest in type II, whereas femoral head cartilage stress retained its progressive type I–III increase. Proximal femoral peak stress remained ordered as type II < type I < type III.

Qualitative contour comparison also showed that the principal pelvic-side high-stress regions remained located at the superior true acetabulum in type I, the true acetabulum–pseudoacetabulum transition region in type II, and the localized pseudoacetabular support region in type III. Thus, the principal classification-related stress-location migration pattern was maintained across the tested bone- and cartilage-modulus conditions.

## Discussion

### Principal findings

The principal finding of this finite element study was that increasing Crowe classification severity across the standardized single-baseline virtual models was associated with spatial migration of the principal high-stress regions rather than a uniform increase in stress across all tissues. Acetabular-side pelvic bone and femoral head cartilage stresses increased from Crowe type I to type III, whereas acetabular/pseudoacetabular cartilage stress peaked in type II. The dominant pelvic-side high-stress region shifted from the superior true acetabulum in type I to the true acetabulum–pseudoacetabulum transition region in type II and then to the localized pseudoacetabular support region in type III.

The loading-sensitivity analysis showed that the principal pelvic-side migration pattern, the type II acetabular/pseudoacetabular cartilage stress peak, and the classification-dependent stress relationships were maintained under the alternative loading condition. Material-property sensitivity analyses likewise produced only small quantitative changes, with absolute relative changes not exceeding 2.46%, and preserved the principal classification-dependent stress relationships and pelvic-side migration pattern. Collectively, these findings support interpreting stress magnitude together with the anatomical location of stress concentration.

### Methodological framework and scope of validation

A methodological feature of this study was the use of a standardized virtual modeling strategy based on a single CT-derived Crowe type I model. By maintaining a common anatomical baseline and identical modeling conditions, this approach reduced inter-individual variability and permitted controlled assessment of how predefined classification-related morphological differences influenced stress distribution and load-transfer pathways.

Mesh-independence analysis and standardized model settings supported numerical stability and internal comparability, but these procedures should be distinguished from experimental validation. The qualitative agreement of the Crowe type I model with experimental observations of rim-directed loading under acetabular undercoverage and with established proximal femoral load-transfer pathways provided external support for anatomical and biomechanical plausibility (Shah et al., 2025; Tachibana et al., 2024). Previous cadaveric studies have also demonstrated that specimen-specific hip finite element models can reproduce experimentally measured cartilage contact mechanics when experimental geometry, loading, and boundary conditions are closely matched (Anderson et al., 2008; Henak et al., 2014).

Nevertheless, the present study did not include patient-matched experimental pressure measurements, *in vivo* joint-force data, or clinical outcome correlation. The external benchmarking therefore does not validate the absolute peak stress values or establish clinical risk thresholds. Moreover, the Crowe type II and III models were generated through predefined morphological modification of the same anatomical baseline rather than reconstructed from independent clinical cases. The model series should consequently be regarded as an internally controlled research framework, and the resulting stress-location patterns as hypothesis-generating biomechanical findings requiring confirmation in independent patient datasets.

### Influence of loading assumptions and omission of muscle forces

The original 600 N loading magnitude was not selected arbitrarily. Body-weight-scale loads of approximately 567–600 N have been used in previous simplified hip finite element models to represent static weight-bearing and to evaluate relative stress-transfer patterns (Zhang et al., 2020; Rudman et al., 2006; Escudier et al., 2018). In particular, previous studies have applied such loads either through the superior pelvis or through the base of the femoral shaft, depending on the model scope and boundary conditions. Nevertheless, a body-weight-level external load should be distinguished from the resultant hip joint force generated after accounting for abductor muscle forces and moment equilibrium.

Accordingly, the baseline 600 N condition was used as a standardized comparative scenario rather than a complete physiological representation of single-leg stance. The additional 1,888 N oblique loading condition was introduced to examine whether the principal findings remained present under a resultant-force magnitude closer to that reported for physiological weight-bearing.

The loading-sensitivity analysis supported the robustness of the principal classification-dependent stress patterns under the tested higher-magnitude oblique loading condition. Acetabular-side pelvic bone and femoral head cartilage stresses retained their type I–III increases, acetabular/pseudoacetabular cartilage stress remained highest in type II, and proximal femoral stress retained the ordering of type II < type I < type III.

Nevertheless, the alternative loading condition should not be interpreted as an explicit simulation of individual muscle forces or dynamic gait. Hip joint loading is influenced by muscle recruitment, and dysplastic anatomy can alter muscle moment arms, lines of action, abductor demand, and the magnitude and direction of the joint reaction force (Song et al., 2020; Harris et al., 2022). These effects may differ among Crowe morphologies because femoral head position, femoral anteversion, and acetabular geometry varied across the standardized models. Consequently, persistence of the principal pelvic-side migration pattern supports robustness to the tested resultant-force scenario but does not eliminate uncertainty associated with omitted classification-specific muscle forces or time-varying gait mechanics.

### Influence of cartilage and material-property simplifications

The material-property sensitivity analyses showed that moderate variation in the assigned bone and cartilage Young’s moduli produced only small quantitative changes in peak stress. Across all tested conditions, relative changes remained within 2.46% of the corresponding baseline values, while the principal classification-dependent stress relationships and pelvic-side stress-location migration pattern were maintained. These findings suggest that the main spatial interpretation was not dependent on a single bone or cartilage modulus within the tested ±20% ranges.

Nevertheless, the sensitivity analysis does not eliminate the limitations associated with the simplified tissue representations. Uniform femoral head and acetabular/pseudoacetabular cartilage thicknesses were applied across all models, and all tissues were represented using homogeneous, isotropic, linear elastic properties. Physiological cartilage exhibits regional thickness variation and nonlinear, time-dependent behavior, while clinical DDH may involve focal cartilage defects, degeneration-related tissue loss, and changes in material properties. Bone also exhibits regional density variation, cortical–cancellous heterogeneity, and anisotropy.

The present one-factor-at-a-time sensitivity analyses did not evaluate interactions among material properties, cartilage thickness, degeneration, contact behavior, and loading conditions. Accordingly, preservation of the principal findings supports robustness within the tested parameter ranges but should not be interpreted as validation of the simplified material formulations or of the absolute peak stress values. Future studies should incorporate MRI- or CT-arthrography-based regional cartilage reconstruction, degeneration-dependent cartilage properties, and CT-density-based heterogeneous bone assignment.

### Acetabular-side biomechanical implications

The acetabular-side findings highlight the mechanical importance of the true acetabulum–pseudoacetabulum transition region in the Crowe type II model. The combination of abnormal curvature, altered contact direction, and limited geometric continuity may contribute to the localized cartilage stress concentration observed in this region. Although acetabular/pseudoacetabular cartilage stress decreased in type III compared with type II, this should not be interpreted as a mechanically safer state because acetabular-side pelvic bone and femoral head cartilage stresses continued to increase. Instead, the lower cartilage peak may reflect further transfer of the dominant load-bearing pathway from the anatomical acetabular cartilage toward pseudoacetabular bony support and displaced femoral head contact. These results demonstrate why peak stress values should be interpreted together with the anatomical location and tissue correspondence of the high-stress region.

### Femoral-side biomechanical implications

Femoral head cartilage stress increased from Crowe type I to type III, accompanied by displacement of the principal contact region toward the superolateral femoral head and the apex contacting the pseudoacetabulum. This pattern suggests that progressive undercoverage and superior displacement reduced the effective contact area and concentrated load within a smaller, displaced region.

More broadly, a recent three-dimensional CT study demonstrated that variations in femoral head–neck morphology and acetabular coverage consistent with femoroacetabular impingement may be present even in asymptomatic adults (İsmailoğlu et al., 2025). Although femoroacetabular impingement and DDH represent different morphological conditions, this finding reinforces the broader principle that osseous morphology should not be interpreted as a direct indicator of clinical disease in isolation; rather, its biomechanical significance depends on how joint shape, coverage, and congruence influence contact location and load transfer. Accordingly, the morphology-related stress patterns observed in the present models should be interpreted as biomechanical findings rather than direct clinical risk thresholds.

Under the baseline loading condition, proximal femoral peak stress was highest in Crowe type III, while the type I value slightly exceeded the type II value. The same ordering of type II < type I < type III was retained under the alternative loading condition. Across both loading scenarios, the principal stress concentration remained located in the femoral neck and medial proximal femoral load-transfer region. Recent anatomical and finite element work by Shah et al. demonstrated that compressive load transfer in the proximal femur is primarily supported by the primary vertical trabecular groups, calcar femorale, and medial cortex, whereas horizontal trabecular groups and the lateral cortex contribute to tensile load transfer (Shah et al., 2025). The stress concentration observed in the femoral neck and medial proximal femur was qualitatively consistent with these anatomical load-transfer pathways. Nevertheless, because the present models represented bone as a homogeneous isotropic continuum and did not explicitly resolve trabecular architecture, this comparison supports anatomical plausibility rather than direct validation.

### Potential clinical relevance and future surgical planning

The present findings suggest that biomechanical assessment of DDH should not rely on peak stress magnitude alone. Stress contour patterns, anatomical location, and tissue correspondence may provide complementary descriptors of classification-related load-transfer changes. In particular, focal loading at the superior acetabular rim, true acetabulum–pseudoacetabulum transition region, and pseudoacetabular support region may help characterize how the dominant load-bearing pathway differs among standardized Crowe morphologies. Sustained stress concentration in the femoral neck and medial proximal femur may also be relevant to bone remodeling or fatigue-related changes, although these potential clinical associations require validation in patient-specific cohorts and longitudinal outcome studies.

From a surgical-planning perspective, the present forward virtual sequence may provide a biomechanical framework for future reverse-design simulations of acetabular correction. Progressively more severe standardized morphologies were generated from a Crowe type I anatomical baseline by modifying superior femoral displacement, lateral center-edge angle, acetabular anteversion, and femoral anteversion. In acetabular reorientation procedures such as periacetabular osteotomy, the corrective direction is conceptually reversed: the acetabular fragment is reoriented to improve femoral head coverage and redirect load toward a broader and more anatomically supported true-acetabular region. This reverse relationship applies specifically to the acetabular coverage and orientation components of the modeling sequence, because PAO does not directly reverse femoral anteversion or superior femoral displacement.

Accordingly, future patient-specific virtual PAO analyses could vary acetabular-fragment orientation to generate candidate lateral center-edge angle and acetabular anteversion targets. Candidate correction strategies could be compared using both peak stress magnitude and the anatomical location of stress concentration. A correction that shifts focal loading away from the superior acetabular rim or true acetabulum–pseudoacetabulum transition region toward a broader true-acetabular load-bearing area may represent a more mechanically favorable reconstruction and could complement conventional radiographic targets (Aitken et al., 2024; Qu et al., 2024; Zou et al., 2013; Wang et al., 2016).

However, the present models did not simulate osteotomy cuts, acetabular-fragment rotation, fixation, postoperative joint congruence, or procedure-specific muscle loading. The findings therefore do not define PAO candidacy, optimal correction angles, clinical intervention thresholds, or direct conversion from Crowe type III to Crowe type I. Rather, stress location should be regarded as a hypothesis-generating biomechanical descriptor that may inform future patient-specific and procedure-specific simulations.

### Limitations

This study has several limitations. First, Crowe type II and III models were generated through standardized morphological modification of a Crowe type I model rather than from independent patient-specific CT datasets. This virtual progression design improved comparability across models and helped isolate classification-related biomechanical changes from inter-individual anatomical variability. However, it also limited the representation of the full anatomical heterogeneity of clinical Crowe type II and III populations. Therefore, the findings should be interpreted as controlled biomechanical patterns of standardized morphological progression rather than population-level stress thresholds. In addition, the predefined angular targets were informed by published morphological ranges rather than derived from independent Crowe type II and III patients. Their combined configuration therefore represents one standardized, classification-consistent scenario among the broad spectrum of possible clinical morphologies. The virtual sequence should also not be interpreted as longitudinal disease progression in the source patient.

Second, both the baseline and alternative loading scenarios were simplified static resultant-force conditions. Although the additional 1,888 N oblique loading analysis demonstrated that the principal pelvic-side stress-location migration pattern remained present under a higher-magnitude and non-axial load, neither scenario explicitly reproduced time-varying gait kinematics, ground-reaction forces, or individual muscle recruitment.

Third, cartilage geometry, material properties, and contact relationships were simplified. Uniform cartilage thicknesses of 2.5 mm for the femoral head and 2.0 mm for the acetabular/pseudoacetabular surfaces were applied across all Crowe models, and all tissues were represented using homogeneous, isotropic, linear elastic properties. Although the ±20% material-property sensitivity analyses produced relative peak-stress changes of no more than 2.46% and preserved the principal classification-dependent stress-location pattern, these analyses did not reproduce regional cartilage-thickness variation, focal defects, degeneration-related tissue loss, nonlinear cartilage behavior, heterogeneous bone density, or material anisotropy. They also did not evaluate simultaneous interactions among material, geometric, contact, and loading assumptions. Therefore, the sensitivity findings support robustness within the tested parameter ranges but do not validate the simplified tissue formulations or establish patient-specific absolute stress magnitudes. Moreover, because von Mises stress was used as a comparative scalar output, the cartilage results should not be interpreted as direct measures of contact pressure or degeneration. Future studies should additionally evaluate contact pressure, principal stress and strain, and time-dependent cartilage behavior.

Fourth, no patient-matched experimental or clinical validation was available. The external benchmarking of the Crowe type I baseline model supported only qualitative anatomical and biomechanical plausibility, while the standardized Crowe type II and III morphologies were not compared with independent patient-specific CT reconstructions. Future studies should therefore include actual Crowe type II and III patient datasets and, where feasible, experimental or *in vivo* measurements of contact pressure, contact area, and joint reaction forces.

## Conclusions

This finite element study suggests that biomechanical differences among Crowe type I–III DDH models are reflected not only by changes in peak stress but also by spatial migration of high-stress regions. Crowe type I retained a mainly true-acetabular loading pattern, Crowe type II showed marked stress concentration at the true acetabulum–pseudoacetabulum transition region, and Crowe type III demonstrated further transfer of load to the localized pseudoacetabular bony support region and the corresponding femoral head contact apex.

These findings support the combined interpretation of peak stress values and stress contour patterns when evaluating DDH biomechanics. This single-baseline virtual modeling approach provides an internally controlled framework for exploring how standardized classification-related morphological differences may influence load-transfer patterns. The findings represent controlled biomechanical patterns generated from standardized virtual models and should not be interpreted as patient-specific predictions, population-level stress thresholds, or evidence of natural longitudinal DDH progression. The principal classification-dependent stress relationships and pelvic-side stress-location migration pattern were maintained under both the alternative loading scenario and the tested material-property variations. The external benchmarking of the Crowe type I model supports the anatomical plausibility of the observed stress-distribution pattern but does not constitute direct experimental validation or establish clinically applicable stress thresholds.

Future studies incorporating independent patient-specific Crowe type II and III imaging datasets, MRI- or CT-arthrography-based regional cartilage reconstruction, degeneration-dependent cartilage properties, muscle-force loading, dynamic gait conditions, and clinical follow-up are needed to validate these classification-related mechanical patterns and clarify their clinical relevance.

## Data Availability

The raw data supporting the conclusions of this article will be made available by the authors, without undue reservation.
