# Supplementary material for: Single-baseline virtual progression modeling of Crowe type I–III developmental dysplasia of the hip: a finite element study of stress-location migration
Source: Front Bioeng Biotechnol. 2026 Aug 19;14:1899897. doi: 10.3389/fbioe.2026.1899897 (PMC13533989; doi:10.3389/fbioe.2026.1899897)
Supplement: Supplementary file 1 [file DataSheet1.pdf]

Supplementary Table S1. Mesh independence analysis of the Crowe type III finite element model.

| **Mesh level** | **Elements (n)** | **Nodes (n)** | **Pelvic bone stress (MPa)** | **Acetabular/pseudoacetabular cartilage stress (MPa)** | **Proximal femur stress (MPa)** | **Femoral head cartilage stress (MPa)** |
|---|---|---|---|---|---|---|
| Coarse | 812,436 | 176,852 | 16.71 | 2.22 | 4.48 | 3.28 |
| Medium | 1,364,728 | 298,417 | 17.22 | 2.31 | 4.61 | 3.42 |
| Fine | 2,186,593 | 471,206 | 17.48 | 2.35 | 4.69 | 3.49 |
| Relative change from medium to fine | — | — | 1.51% | 1.73% | 1.74% | 2.05% |

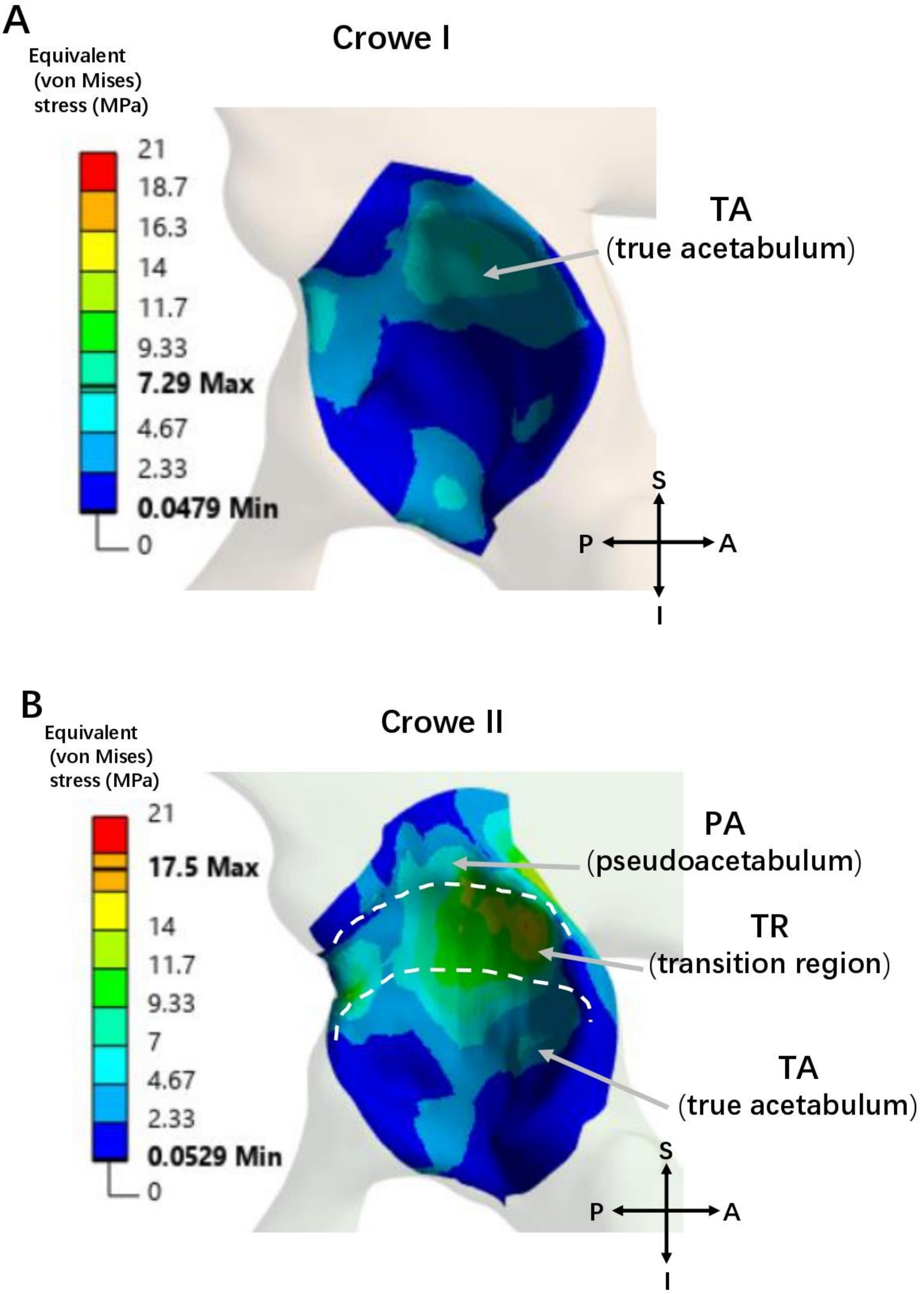
A
Crowe I
Equivalent (von Mises) stress (MPa)
21
18.7
16.3
14
11.7
9.33
7.29 Max
4.67
2.33
0.0479 Min
0
TA
(true acetabulum)
S
P
A
I
B
Crowe II
Equivalent (von Mises) stress (MPa)
21
17.5 Max
14
11.7
9.33
7
4.67
2.33
0.0529 Min
0
PA
(pseudoacetabulum)
TR
(transition region)
TA
(true acetabulum)
S
P
A
I

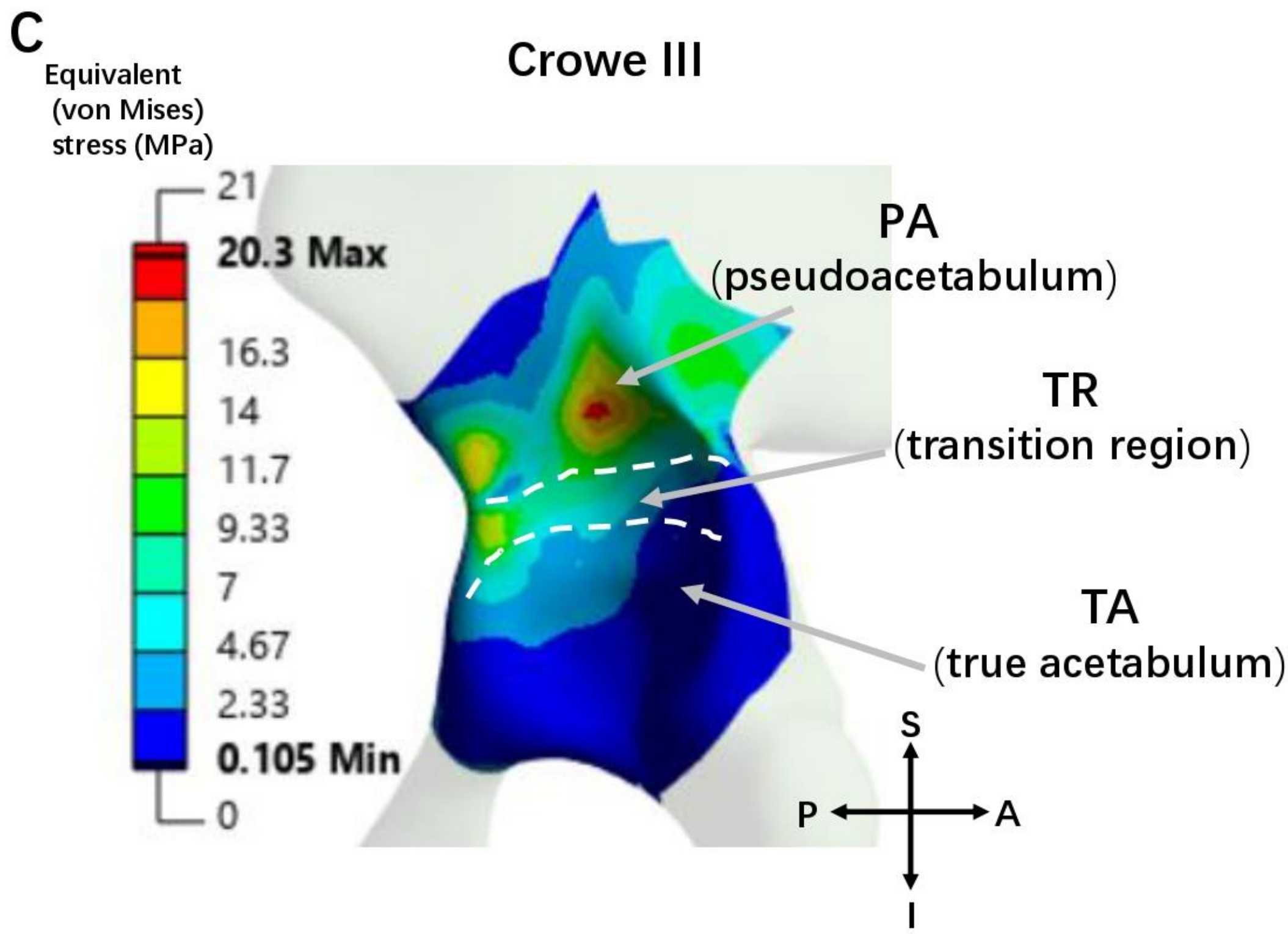
C
Crowe III
Equivalent
(von Mises)
stress (MPa)
21
20.3 Max
16.3
14
11.7
9.33
7
4.67
2.33
0.105 Min
0
PA
(pseudoacetabulum)
TR
(transition region)
TA
(true acetabulum)
S
P
A
I

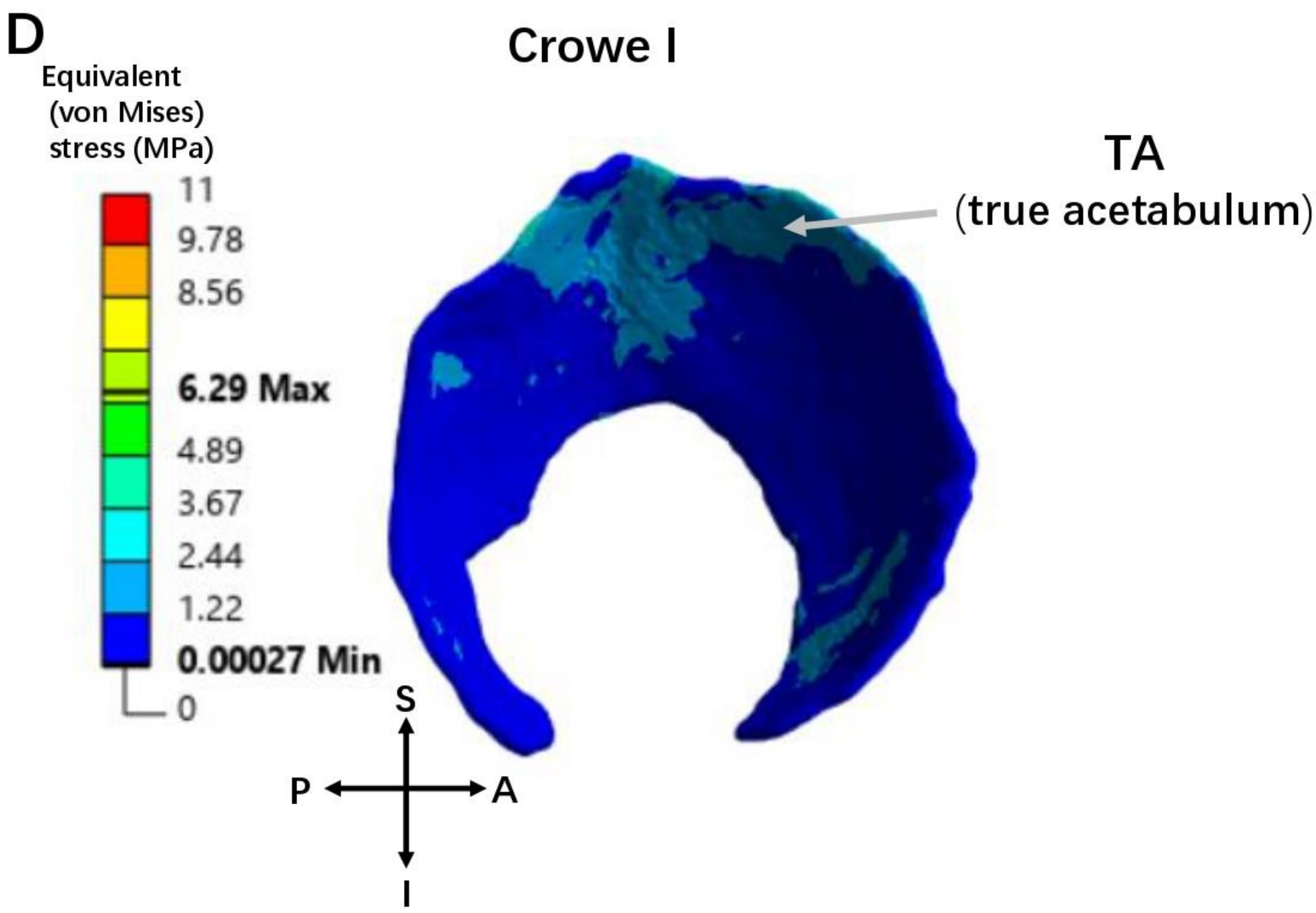
D
Crowe I
Equivalent
(von Mises)
stress (MPa)
11
9.78
8.56
6.29 Max
4.89
3.67
2.44
1.22
0.00027 Min
0
TA
(true acetabulum)
S
P
A
I

E

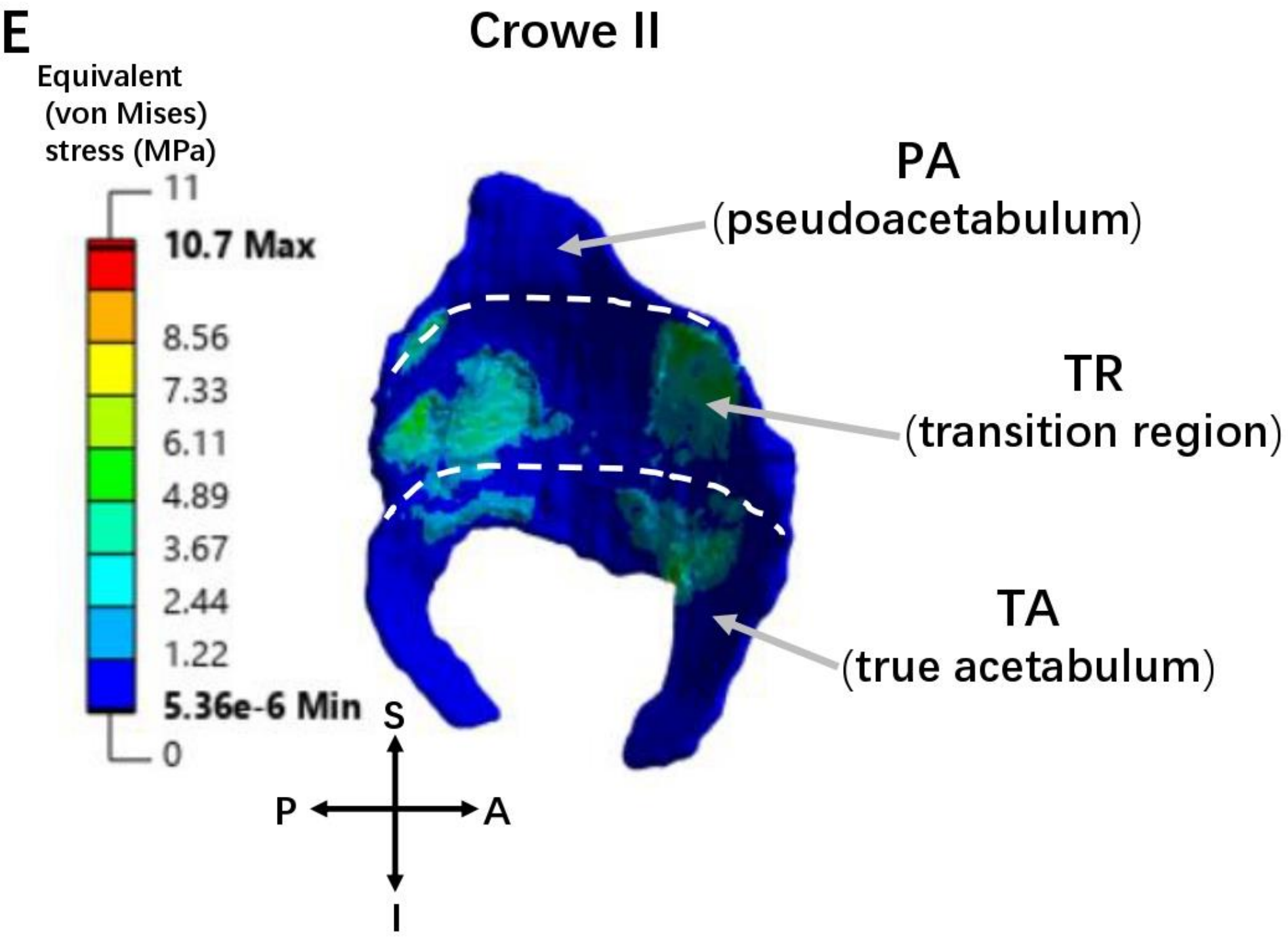


F

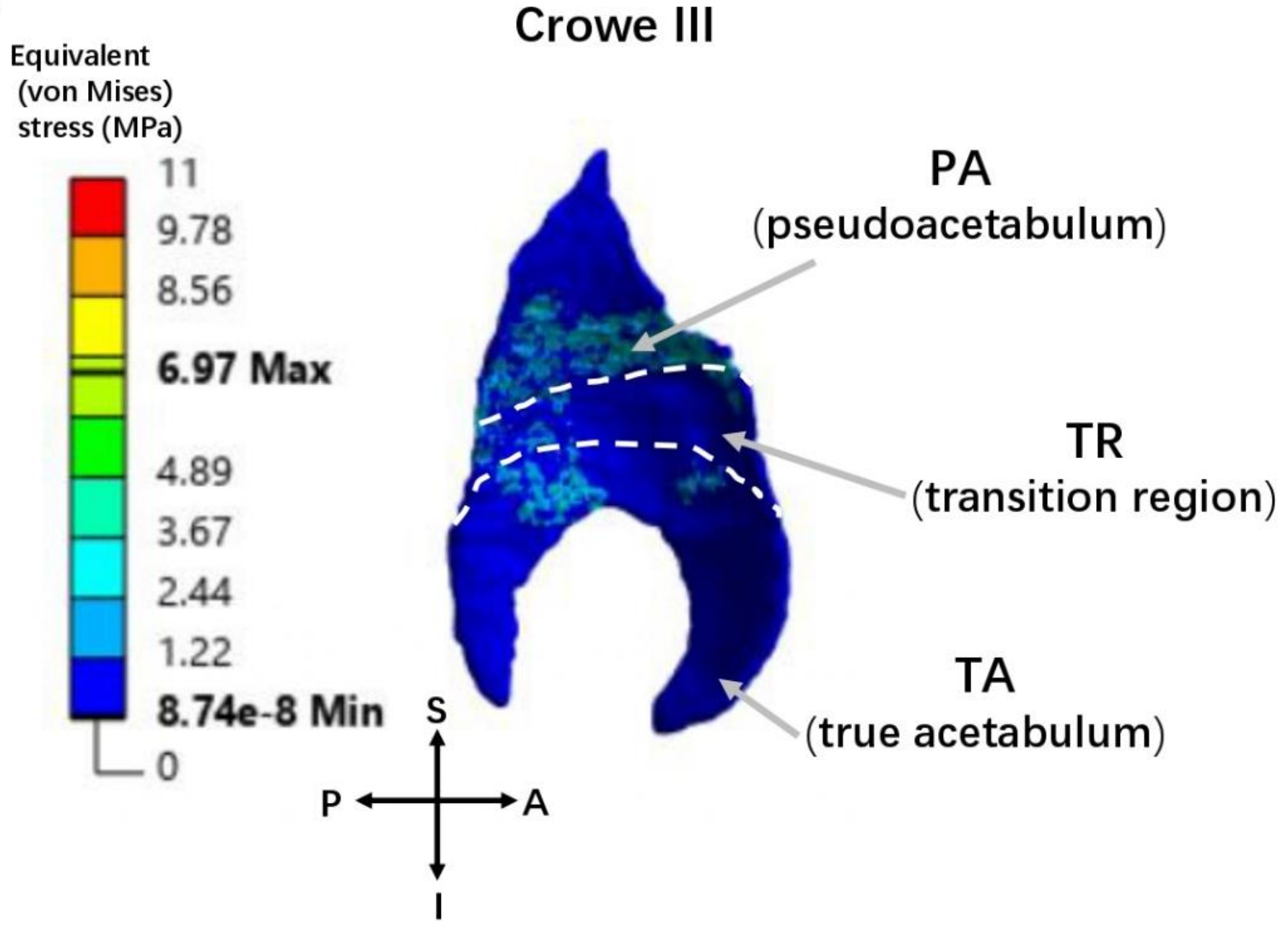


**Supplementary Figure S1. Stress distributions under the alternative 1,888 N oblique loading condition.**

**(A–C)** Acetabular-side pelvic bone stress distributions in the Crowe type I, II, and III models, respectively. An identical color scale of 0–21 MPa was applied across panels A–C. **(D–F)** Acetabular/pseudoacetabular cartilage stress distributions in the Crowe type I, II, and III models, respectively. An identical color scale of 0–11 MPa was applied across

panels D–F. Standardized viewing orientations were used within each tissue group. TA, true acetabulum; TR, true acetabulum–pseudoacetabulum transition region; PA, pseudoacetabulum; S, superior; I, inferior; A, anterior; P, posterior.
